# *Chasteberry* extract alleviates rotenone-triggered testicular injury in rats by modulating oxidative stress, inflammation, apoptosis, and mitochondrial dysfunction

**DOI:** 10.1038/s41598-026-46954-4

**Published:** 2026-04-17

**Authors:** Hebatallah M. Saad, Neveen R. Ashoura, Alyaa R. Salama, Lamiaa G. Wasef, Attaa M. Abd Elrehim, Bothaina H. Essa, Nehad A. Saleh, Ahmed Elsawasany, Amal A. Awad

**Affiliations:** 1 Department of Pathology, Faculty of Veterinary Medicine, Matrouh University, Mersa Matruh, Egypt; 2 Department of Veterinary Pharmacology, Faculty of Veterinary Medicine, Alexandria University, Alexandria, Egypt; 3 Department of Pathology, Faculty of Veterinary Medicine, Alexandria University, Alexandria, Egypt; 4 Department of Pharmacology and Therapeutics, Faculty of Veterinary Medicine, Damanhour University, Damanhour, 22511 Egypt; 5 Department of Physiology, Faculty of Veterinary Medicine, Damanhour University, Damanhour, Egypt; 6 Department of Animal Wealth Development, Faculty of Veterinary Medicine, Damanhour University, Damanhour, 22511 Egypt; 7 Department of Animal Hygiene, Faculty of Veterinary Medicine, Damanhour University, Damanhour, Egypt; 8 Department of Forensic Medicine and Toxicology, Faculty of Veterinary Medicine, Alexandria University, Alexandria, Egypt

**Keywords:** *Chasteberry* extract, Rotenone, Oxidative stress, Apoptosis, Testes, Rats, Biochemistry, Cell biology

## Abstract

Widespread usage of the pesticide rotenone has been linked to several health issues in both people and animals. Thus, we aimed to counteract rotenone-induced testicular oxidative damage using *Chasteberry* extract (CBE), while concurrently investigating the underlying mechanisms. Forty male albino rats were randomized into four groups (10 rats per each group): served as control (received 1 mL of saline orally, and 2% dimethyl sulfoxide (DMSO) at a dose of 10 mL/kg for 3 times/week, intraperitoneally), CBE (165 mg/kg/daily, orally), rotenone (3 mg/kg/3times/week, intraperitonially), and combination (CBE and rotenone). Treatments were administered for 60 consecutive days. Our data indicated that rotenone injection resulted in hormonal imbalance, oxidative damage, inflammation, apoptosis, as well as mitochondrial dynamic dysfunction in testicular tissue. While the co-administration of CBE with rotenone significantly mitigated these deleterious effects and restored sperm morphology and count, as evidenced biochemically by upregulation in follicle-stimulating hormone, luteinizing hormone and testosterone hormonal levels. Also, the CBE-supplemented rotenone group showed enhanced antioxidant enzyme activities (superoxide dismutase, catalase, total and reduced glutathione) and reduced malondialdehyde levels, oxidized glutathione, and proinflammatory cytokine levels (tumor necrosis factor and interleukin 1β). These results were further confirmed by histopathological investigation, which revealed marked restoration of testicular histoarchitecture with reduction in nuclear factor kappa B and caspase 3 immunoreactivity in the combination group. On the molecular level, CBE supplementation restored the antioxidant mRNA transcript of antioxidant genes, mitochondrial biogenesis genes, and mitochondrial dynamics, as evidenced by upregulation in mitofusin and downregulation in dynamin-related protein 1. In conclusion, CBE is a promising protective agent against rotenone-induced testicular damage via its antioxidant, anti-inflammatory, antiapoptotic, and mitochondrial biogenesis-enhancing effects.

## Introduction

Rotenone is a natural isoflavone botanical insecticide derivative of plants in the Fabaceae family, such as Derris elliptica, *Lonchocarpols spp*., and *Tephrosia villosa spp*^1–3^. Historically, it has been used as a fish poison in Southeast Asia and South America, and it is now widely applied as an insecticide and pesticide in agriculture and pest management practices^4^. Despite its organic origin and effectiveness, rotenone raises significant concerns due to environmental contamination and potential health impacts^5,6^. Additionally, it can affect food webs and cause secondary poisoning by impacting terrestrial species exposed to contaminated soil or water sources^7,8^. Prolonged exposure to rotenone has been related to numerous health problems in both humans and animals. Its neurotoxicity, cognitive impairment, and behavioral changes are particularly concerning^9,10^. Other health implications have also been reported, including endocrine disruption^11^, reproductive toxicity, and teratogenicity^12,13^, systemic toxicity^14^, metabolic disorders^15^, gastrointestinal issues^16^, cardiovascular effects^17^, hepatotoxicity^18^, and nephrotoxicity^19^. Rotenone exposure is strongly associated with reproductive toxicity and endocrine disruption in both humans and various animal species^20,21^. The testicular damage induced is linked to rotenone’s ability to induce oxidative stress by producing reactive oxygen species (ROS)^22^, which interfere with mitochondrial function during spermatogenesis^23^, leading to DNA damage of sperm, altered morphology, and reduced sperm count and quality^24^. These ROS also cause structural damage to testis cells, including degeneration, inflammation, atrophy, or even germ cell apoptosis^25^. Furthermore, ROS impairs Leydig cells^26^, decreasing testosterone production, which affects secondary sexual characteristics, libido, and mating behavior^27^. Rotenone also disrupts the endocrine system by interfering with the regulation of testosterone, follicle-stimulating hormone (FSH), and luteinizing hormone (LH)^27^, crucial hormones for testicular function and fertility^21^. These reproductive consequences underscore the importance of assessing rotenone’s endocrine-disrupting potential, especially given its widespread agricultural use^28^.

Under physiological conditions, apoptosis, a regulated process of programmed cell death, is essential for maintaining the correct number of cells and ensuring the stability of tissues^29^. According to recent research, increased apoptosis of Leydig cells may be one of the main mechanisms behind the decrease in testosterone synthesis resulting from environmental contaminants^30^. There is tow crucial molecules linked to cell death are the proapoptotic BAX and the antiapoptotic BCL2^31^. The pro-apoptotic BCL2-associated X, apoptosis regulator (BAX) protein is increased under oxidative stress and reduces membrane permeability^32^. On the other hand, BCL2 promotes cell survival by preventing the effects of the proapoptotic protein BAX, hence preserving the integrity of the mitochondrial membrane^33^. Furthermore, mitochondrial dynamics can be identified as the process of mitochondrial fusion or division. Mitochondrial structure, distribution, and function rely heavily on proteins encoded by nuclear DNA. Defects in these proteins cause problems with how mitochondria move and change shape, a process known as mitochondrial dynamics. Mitochondrial fusion and fission can be monitored by mitofusin 2 (MFN2) and dynamin-related protein 1 (DRP1), two dynamin family members^34^.

In response to rotenone’s environmental and health impacts, several strategies are being developed to regulate or phase out its use, favoring safer pest control methods. One of these strategies is utilizing antioxidants from sources like herbal remedies to counteract rotenone-induced testicular toxicity, where they are being explored for their ability to neutralize ROS and reduce oxidative damage^35,36^. Among the most often used therapeutic herbs *is Chasteberry* (*Vitex Agnus-Castus*), which belongs to the *Lamiaceae* family, originating in the Mediterranean region and spreading over Europe, Asia, and North Africa^37^. It is one of the most prevalent plants with a high concentration of flavonoids, which protect cells against oxidative stress by enhancing the balance between oxidants and antioxidants^38^*. Chasteberry* is gaining interest for improving male reproductive health in animals^39^, building on its widespread application in women’s reproductive health^40^. *Chasteberry* has been studied in livestock for its potential to enhance breeding by improving spermatogenesis and balancing hormones crucial for testicular function^39,41^. Its bioactive compounds (flavonoids, terpenoids, iridoids) influence hormonal pathways like prolactin and gonadotropins, essential for regulating reproductive function in both sexes^42,43^. Furthermore, recent research by Olaolu, et al.^44^ suggests that *Chasteberry* ameliorated the cadmium- induced oxidative stress in testicular tissues by improving the levels of antioxidants as catalase and superoxide dismutase (SOD), as well as sex hormones (LH, testosterone).

Therefore, this study was planned to evaluate the toxicological impacts of rotenone on the testes of male rats and the efficacy of *Chasteberry* extract (CBE) supplementation in mitigating rotenone-induced testicular toxicity and illustrating the potential underlying mechanisms.

## Materials and methods

### Experimental design

A total of forty adult male albino rats that appeared to be in good health, weighing between 200 and 220 g at the age of 8 weeks, were used in the experiment. Rats were acquired from the Medical Research Institute, Alexandria University, and housed in the laboratory of the Pharmacology Department, Faculty of Veterinary Medicine, Damanhour University. Rats were housed in well-ventilated polycarbonate cages with sawdust bedding, with 10 rats per cage, Maintained at 21 ± 2 °C with 60 ± 2% humidity under a natural light–dark cycle. The bedding is replaced every day to ensure a clean and dry environment. The rats were free from specific pathogens with unlimited access to a conventional diet and water. Before starting the experiment, the rats were adapted for two weeks.

**Fig. 1:**
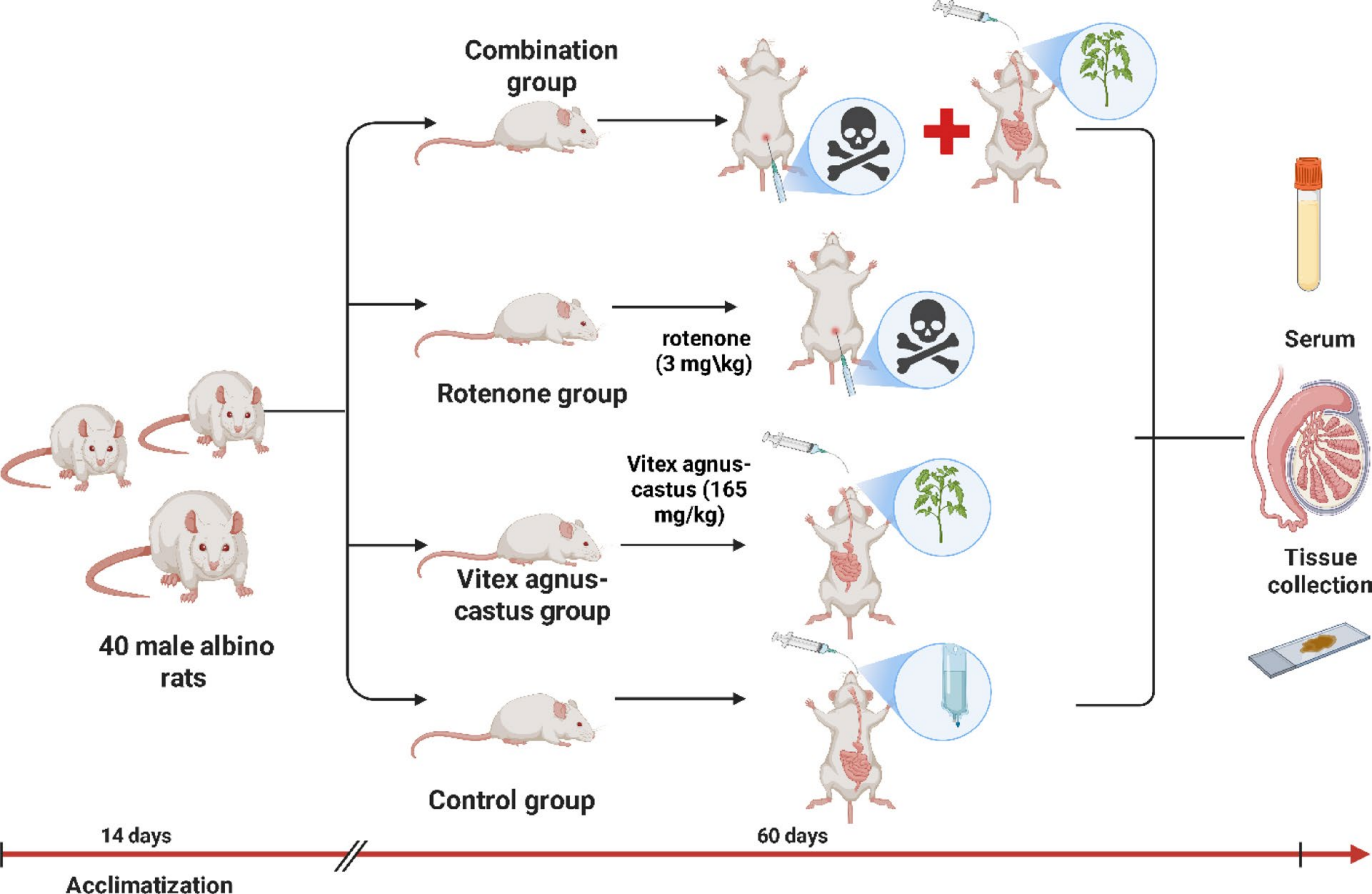
Experimental design.

Forty male albino rats were randomly allocated to four experimental groups using a computer-generated random number sequence, the sample size (n = 10 per group) was chosen based on previous studies using similar experimental models and endpoints. Group 1, serving as the control, was given 1 mL of saline orally, and 2% dimethyl sulfoxide (DMSO) was administered at a dose of 10 mL/kg for 3 times/week, intraperitoneally. Group 2 (Control CBE group) received *Chasteberry* extract (CBE) (previously prepared as described in our earlier study by Saad et al.^45^) was administered at a dosage of 165 mg/kg orally^46^ for 2 months. Group 3 (Rotenone group) received an intraperitoneal injection of rotenone (Sigma, St., Louis, MO, USA) at a dose of 3 mg/kg dissolved in 2% DMSO for 3 times/week for 2 months^47,48^. Group 4 (Combination group) received both CBE and rotenone at the previous doses for 2 months (Fig. 1).

The experiment has been approved by the Institutional Animal Care and Use Committee (IACUC) of Damanhour University under permission number DMU/VetMed-march-09-2025/009. All methods were performed in accordance with the relevant guidelines and regulations. Every technique followed the guidelines set out by ARRIVE^49^.

### Sampling

At the end of the trial period, retro-orbital blood was collected in plain tubes under light isoflurane anesthesia which performed via the medial canthus using heparin-coated capillary tubes. The blood samples were left to coagulate for 30 min at 25 °C, and then centrifuged for 15 min to separate the serum. After that, the serum layer was separated into a clean Eppendorf tube for further biochemical analysis. Then, the rats were euthanized with cervical dislocation under complete isoflurane anesthesia, and their tests with epididymis were carefully dissected. The right one was fixed for histopathological and immunohistochemical analysis in 10% neutral buffered formalin. The left testes were quickly excised and homogenized for further biochemical and gene expression analysis.

### Serum LH, FSH, and testosterone levels assessment

Serum levels of follicle-stimulating hormone (FSH), luteinizing hormone (LH), and testosterone were measured using commercial ELISA kits (CUSABIO Co., Catalog Nos. CSB-E12654r, CSB-E06869r, and CSB-E05100r, respectively), following the protocols provided by the manufacturer. The procedures were conducted in accordance with standard immunoassay principles as described by Tietz^50^. No modifications were made to the manufacturer’s recommended protocol. Before the assay, all samples and standards were allowed to reach room temperature to ensure uniform reaction conditions. To preserve cell and tissue integrity, samples were washed using phosphate-buffered saline (PBS) warmed to 37 °C. Samples and standards were assayed in duplicate. For LH and testosterone, 50 µL of sample was added to each well, followed by 50 µL of HRP-conjugated hormone and 50 µL of specific antibody, then incubated for 1 h at 37 °C. For FSH, 100 µL of sample was added, followed by 100 µL of biotin-conjugated antibody and 100 µL of HRP-avidin, with incubations of 2 h and 1 h at 37 °C, respectively. Wells were washed according to kit instructions between each incubation. Substrate solutions were added and incubated in the dark at 37 °C (15–30 min), and the reaction was stopped with Stop Solution. Optical density was read at 450 nm using a microplate reader, with wavelength correction at 540–630 nm as applicable. Standard curves were constructed in duplicate and used to calculate sample concentrations using a four-parameter logistic (4-PL) curve-fit, multiplying by any dilution factor when necessary. The detection ranges were 0.3–60 mIU/mL for LH, 0.156–10 ng/mL for FSH, and 0.13–25.6 ng/mL for testosterone, with sensitivities of 0.15 mIU/mL, 0.039 ng/mL, and 0.06 ng/mL, respectively.

### Testicular oxidative and antioxidant biomarkers assessment

Testicular malondialdehyde (MDA) was quantified by the method of Draper and Hadley^51^. Superoxide dismutase (SOD) and catalase (CAT) activities were assessed according to the techniques outlined by Flohé and Günzler^52^, Beers and Sizer^53^, respectively. While, total glutathione (tGSH), reduced (GSH) and oxidized glutathione (GSSG) contents were estimated^54^. All prior parameters were measured spectrophotometrically according to the manufacturer’s directions using commercial kits from Bio-diagnostic Co., Egypt. Protein concentration was determined using the Bradford protein assay, and all biochemical measurements were normalized to protein content.

### Sperm parameters measurements

Sperm samples were collected from the caudal region of the epididymis (approximately 1 cm from the distal end) and minced in 2 mL of phosphate-buffered saline (PBS). The suspension was incubated at 37 °C for 30 min to facilitate spermatozoa dispersion. Following incubation, the solution was gently agitated to ensure homogenization. Sperm count, motility, and morphological abnormalities were then assessed using a light microscope at 400× magnification^55^. All scoring and assessments were performed by observers blinded to the experimental group allocation, in order to minimize observer bias.

### Sperm counting

A 10 µL of the diluted sperm suspension was loaded into each chamber of a Neubauer hemocytometer (LABART, Darmstadt, Germany), and total sperm count was determined by counting cells within 250 small squares^56^.

### Sperm motility

Sperm motility was evaluated by randomly selecting four different microscopic fields per sample, with a total of 200 spermatozoa examined per slide to calculate the percentages of motile and non-motile cells^55^.

### Sperm abnormality

Sperm morphological abnormalities were assessed using light microscopy according to established criteria. Abnormalities were classified into head, midpiece, and tail defects. Head abnormalities included amorphous, banana-shaped, double-headed, or detached heads; midpiece abnormalities included bent or irregular midpieces and retained cytoplasmic droplets; and tail abnormalities included coiled, bent, short, or double tails. At least 200 spermatozoa per animal were examined, and results were expressed as the percentage of abnormal sperm. Sperm smears were stained using eosin–nigrosine, and morphological evaluation was performed under oil immersion at ×1000 magnification^57,58^.

### Testicular proinflammatory cytokines and apoptotic markers assessment

Tumor necrosis factor-alpha (TNF-α) was quantified in testicular homogenates using a rat TNF-α ELISA kit (Chongqing Biospes Co., Ltd.; Cat. No. BEK1214). All assays were performed in duplicate, and the mean of the two readings was used for analysis. Sample dilutions were adjusted according to the expected concentration to fall within the kit’s detection range (tissue lysates: 15.6–1000 pg/mL; sensitivity < 1 pg/mL). Biotin-conjugated anti-TNF-α secondary antibody was used at a 1:100 dilution and incubated for 60 min at 37 °C, followed by incubation with Avidin–Biotin-Peroxidase Complex (1:100) for 30 min at 37 °C. TMB substrate was applied for approximately 30 min at 37 °C in the dark, and the reaction was stopped with acidic stop solution. Absorbance was read at 450 nm using a microplate reader. Standard curves were constructed using manufacturer-provided standards and validated according to the kit instructions.

Interleukin-1β (IL-1β) levels in testicular homogenates were measured using a rat IL-1β ELISA kit (CUSABIO Co., Ltd.; Cat. No. CSB-E08055r). All samples and standards were assayed in duplicate. Samples were diluted with the provided Sample Diluent as needed to fall within the kit’s detection range (0.156–10 ng/mL; sensitivity < 0.039 ng/mL). Biotin-conjugated anti-IL-1β antibody was applied as a 1× working solution and incubated for 1 h at 37 °C, followed by incubation with HRP-avidin (1x) for 1 h at 37 °C. TMB substrate was added for 15–30 min at 37 °C in the dark, and the reaction was stopped with acidic stop solution. Absorbance was read at 450 nm using a microplate reader, and concentrations were calculated by interpolation from a standard curve constructed with the provided standards.

Apoptosis regulator BAX levels in testicular homogenates were measured using a rat BAX ELISA kit (CUSABIO Co., Ltd.; Cat. No. CSB-EL002573RA). All samples and standards were assayed in duplicate. Samples were diluted with the provided Sample Diluent as needed to fall within the kit’s detection range (62.5–4000 pg/mL; sensitivity < 15.6 pg/mL). Biotin-conjugated anti-BAX antibody was applied as a 1× working solution and incubated for 1 h at 37 °C, followed by incubation with HRP-avidin (1x) for 1 h at 37 °C. TMB substrate was added for 15–30 min at 37 °C in the dark, and the reaction was stopped with acidic stop solution. Absorbance was read at 450 nm using a microplate reader, and concentrations were calculated by interpolation from a standard curve constructed with the provided standards.

B-cell CLL/lymphoma 2 (BCL2) levels in testicular homogenates were measured using a rat BCL2 ELISA kit (CUSABIO Co., Ltd.; Cat. No. CSB-E08854r). All samples and standards were assayed in duplicate. Samples were diluted with the provided Sample Diluent as needed to fall within the kit’s detection range (0.312–20 ng/mL; sensitivity < 0.078 ng/mL). Biotin-conjugated anti-BCL2 antibody was applied as a 1× working solution and incubated for 1 h at 37 °C, followed by incubation with HRP-avidin (1x) for 1 h at 37 °C. TMB substrate was added for 15–30 min at 37 °C in the dark, and the reaction was stopped with acidic stop solution. Absorbance was read at 450 nm using a microplate reader, and concentrations were calculated by interpolation from a standard curve constructed with the provided standards.

### Histopathologic assessments

The traditional paraffin embedding method was used to handle fixed testicular tissues. The sections were cut into 4 µm-thick slices and stained using Hematoxylin and Eosin (H&E) and Periodic Acid–Schiff (PAS) techniques^59,60^. Cosentino’s pathologic score^61^ and the mean Johnsen histologic score^62^ were used to assess the extent of testicular damage and the efficiency of spermatogenesis, respectively. The testis is classified into four categories according to Cosentino’s grading system, which ranges from normal testicular parenchyma to necrotic tissue A score of 10 in JTBS shows normal spermatogenesis, a score of 9 indicates that there are many spermatozoa scattered throughout the germinal epithelium, and a score of 8 suggests that there are few spermatozoa. On the other hand, a score of 1 denotes the complete lack of cellular structure inside the seminiferous tubules, and a score of 7 to 2 signals the end of maturation.

### Immunohistochemical protein assay

To aid in antigen retrieval, the slides were deparaffinized, rehydrated, and then submerged in a 10 mM sodium citrate buffer solution, they were then heated in a microwave. Washing with PBS was done after the endogenous peroxidase enzyme was inactivated by incubating it with 3% hydrogen peroxide in absolute methanol for 30 min at 4 °C. By using a 10% normal blocking serum for 60 min at room temperature, nonspecific binding was successfully prevented. After being treated against nuclear factor kappa beta (NF-κB p65, ab16502, 1:200, Abcam) and caspase-3 (ab32351, 1:100, Abcam) overnight at 4 °C, the first antibodies were cleaned with PBS and incubated for 60 min with a second antibody (Santa Cruz Biotechnology, Dallas, TX, USA). After being bathed with PBS, the slices were then treated with the streptavidin-peroxidase conjugate for 30 min. After that, the streptavidin–biotin complex was incubated for three minutes at 7.0 pH in a 3, 3′-diaminobenzidine tetrahydrochloride-H2O2 solution. After that, the slices were dyed with Mayer’s hematoxylin 47 after being carefully cleaned with distilled water^63^. Using FIJI ImageJ software (NIH, USA) at a fixed magnification of ×400, the area% of NF-κB and caspase-3 immune expression was measured in ten haphazardly chosen areas from six animals in each group. All measurements and scoring were performed by observers blinded to the experimental groups to ensure unbiased assessment.

### Quantitative real-time PCR (qPCR) analysis

#### Total RNA isolation

Freshly obtained testicular samples were collected and kept in liquid nitrogen till RNA extraction. Total mRNA was secluded from the collected samples via the RNeasy Mini Kit (Qiagen, Germany) following manufacturer’s directions.

#### Reverse transcription and cDNA synthesis

RNA samples were converted into cDNA using the TOPscript™ RT DryMIX (dT18/dN6 plus) kit (Enzynomics Co Ltd, Korea, cat number RT220), following the manufacturer’s protocol. Following extraction, the extracted RNA was checked for integrity via 2% agarose gel electrophoresis and quantified with a Nanodrop (Genova spectrophotometer 377501, Jenway, Vernon Hills, IL).

The expression levels of peroxisome proliferator-activated receptor-gamma coactivator-1 Alpha (PGC-1α), mitochondrial transcription factor A (Tfam), Dynamin-related protein 1 (DRP1), Heme oxygenase 1 (HO-1), NAD (P)H quinone dehydrogenase 1 (NQO1), and Mitofusin 2 (MFN2) in tissue samples were quantitatively assessed. This was achieved using the ViPrime PLUS Taq qPCR Green Master Mix I (Vivantis Technologies, Malaysia, cat. number QLMM12) in conjunction with gene-specific primer sets. (Table 1). The reaction mix was prepared as follows: 10 µl ViPrime PLUS Taq qPCR Green Master Mix I. 1 µl forward primer (20 pmol), 1 µl reverse primer (20 pmol), cDNA, and the volume was completed to 25 µl with RNase-free water. The Bio-Rad CFX connect thermal cycler was programmed according to the cycling conditions as follows: Initial Activation at 95 °C for 10 min, 40 cycles of denaturation at 94 °C for 15 s, annealing at 55 °C for 15 s, and Extension at 60 °C for 15 s. Melt curve analysis was performed at the end of each qPCR run to verify the specificity of the amplified products. Single sharp peaks were observed for all target genes, indicating the absence of non-specific amplification or primer-dimer formation. For relative quantification, target gene expression was measured relative to the 18S rRNA reference gene, utilizing the comparative cycle threshold (2^−ΔΔCt^) method^64^.

**Table 1:** Primers used for the qRT-PCR amplification.

Gene	Accession number	Primer sequence	
18S rRNA (Reference gene)	NR_046237.2	F	GTAACCCGTTGAACCCCATT
		R	CAAGCTTATGACCCGCACTT
PGC-1α	NM_031347.1	F	GTGCAGCCAAGACTCTGTATGG
		R	GTCCAGGTCATTCACATCAAGTTC
TFAM	NM_031326.2	F	CCCACAGAGAACAGAAACAG
		R	CCCTGGAAGCTTTCAGATACG
HO-1	NM_012580.2	F	CGTGCAGAGAATTCTGAGTTC
		R	AGACGCTTTACGTAGTGCTG
NQO1	NM_017000.3	F	GCCGAACACAAGAAGCTGGAAG
		R	GGCAAATCCTGCTACGAGCACT
DRP1	NM_053655.3	F	GATGCCATAGTTGAAGTGGTGAC
		R	CCACAAGCATCAGCAAAGTCTGG
MFN2	NM_130894.4	F	GCCAGCTTCCTTGAAGACAC
		R	GCAGAACTTTGTCCCAGAGC

#### Quantifying of the mitochondrial genomes per a single cell

An RT-qPCR assay was employed to assess the mtDNA copy number in experimental samples, specifically by measuring the ratio between mitochondrial and nuclear PGC-1α gene PCR amplicons. The total genomic DNA required for this assay was isolated using the DNeasy® Blood and Tissue Kit (Qiagen, Germany, catalog number: 69504), as per the manufacturer’s instructions. The primer set for mtDNA were as follows: forward 5′-ACACCAAAAGGACGAACCTG-3′ and reverse 5′-ATGGGGAAGAAGCCCTAGAA-3′; and for PGC1α gene, forward 5′-ATGAATGCAGCGGTCTTAGC-3′ and reverse 5′-AACAATGGCAGGGTTTGTTC-3′. Reactions were carried out using SYBR Green PCR Master Mix (Applied Biosystems; Thermo Fisher Scientific, Inc.), 0.5 µM forward and reverse primer, and 50 ng genomic DNA were used with the following conditions: 95˚C for 10 min followed by 40 cycles of 95˚C for 15 s, 60˚C for 30 s and 72˚C for 30 s^65^. To determine the quantity of mitochondrial DNA (mtDNA) compared to nuclear DNA, scientists measured the levels of mtDNA relative to a nuclear gene called PGC-1α within the same sample. This was done by calculating and normalizing the threshold cycles (Ct values) of the mtDNA against those of PGC-1α using 2 ^−ΔΔCt^ method^64^.

First, the threshold cycle number values (Ct value) for the genes were calculated using SDS software v 1.7 (Applied Biosysstems), then Ct values of the mtDNA against those of PGC-1α were calculated using the following equation, RC = 2^ΔCt^, where RC is the calculated relative copy number and ΔCt is CtPGC-1α- CtmtDNA^66^.

Several lines of evidence indicate that the transcriptional coactivator PGC-1α serves as a key regulator of mitochondrial biogenesis in mammals. The transcriptional coactivator γ coactivator 1α (PGC 1α) regulates genes that are associated with energy metabolism. PGC 1α interacts with the nuclear peroxisome proliferator-activated receptor (PPAR) that permits the interaction of PGC 1α with a number of transcription factors^67^.

### Statistical analysis

Data are shown as mean ± SEM. Statistical evaluation was carried out using GraphPad Prism (version 9.0; GraphPad Software, San Diego, CA, USA). Normality of the data distribution was checked using the Shapiro–Wilk test. Group differences were examined by one-way ANOVA, followed by Tukey’s multiple comparison test when appropriate. Statistical significance was accepted at *p* < 0.05.

## Results

### Effect of *Chasteberry* extract (CBE) against rotenone-induced serum FSH, LH, and testosterone levels alterations in rats’ testes

**Fig. 2:**
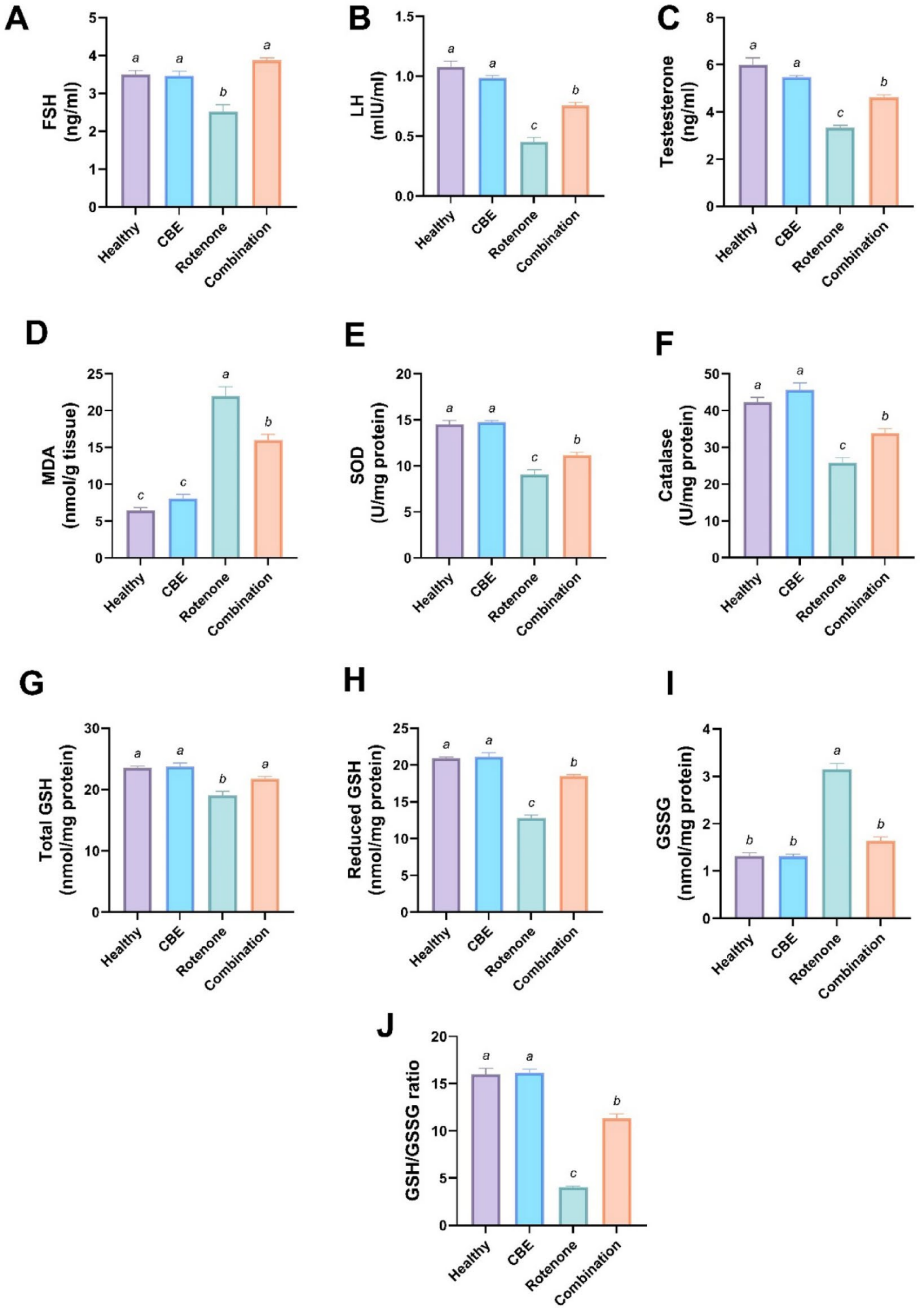
Effect of *Chasteberry* extract (CBE) against rotenone-induced hormonal and redox imbalance in rats’ testes. (**A**) Follicle-stimulating hormone (FSH, ng/ml). (**B**) Luteninzing hormone (LH, mlU/ml). (**C**) Testosterone (nglml). (**D**) Malondialdehyde (MDA, nmol/g tissue). (**E**) Superoxide dismutase (SOD, U/mg). (**F**) Catalase (U/mg). (**G**) Total glutathione (Total GSH, nmol/mg protein). (**H**) Reduced glutathione (reduced GSH, nmol/mg protein). (**I**) Oxidized glutathione (GSSG, nmol/mg protein). (**J**) Reduced glutathione/oxidized glutathione (GSH/ GSSG) ratio. Data expressed as Mean ± SE (n = 5). Data was analyzed using one-way ANOVA followed by Tukey’s post hoc test. Bars bearing different letters are significantly different at *p* < *0.05*.

As shown in Fig. 2A–C, sex hormones (FSH, LH, and testosterone) exhibited no-significant changes between the healthy rats and CBE-treated rats. However, the rotenone-intoxicated group displayed a significant *(p* < *0.05)* decline in serum FSH, LH, and testosterone levels by 27.81%, 58.07%, and 44.3%, respectively, versus the healthy group. Whereas, the combination group displayed a significant *increase (p* < *0.05)* in levels of FSH, LH, and testosterone by 53.53%, 66.81%, 38.60%, respectively relative to the rotenone-intoxicated rats.

### Effect of *Chasteberry* extract (CBE) against rotenone-induced testicular oxidative/antioxidant biomarkers alterations in rats’ testes

As displayed in Fig. 2D, no-significant differences were observed between healthy rats and CBE-treated rats in MDA levels. Furthermore, a significant increase *(p* < *0.05)* in the MDA concentration was detected in rotenone-treated rats by 239.51% in contrast to the control healthy group. While, the combined treatment of CBE with rotenone exhibited a significant *(p* < *0.05)* decline in MDA level by 47.97% relative to the rotenone group.

As showed in Fig. 2E–H, I, no significant changes were reported between healthy rats and CBE-treated rats in SOD and CAT as well as, total GSH, reduced GSH and GSH/GSSG ratio levels. Conversely, these measured parameters demonstrated a statistically significant decrease *(p* < *0.05)* in the rotenone-intoxicated group (by 37.54%, 38.75%, 19.13%, 39.03%, and 74.61%, respectively) as relative to the healthy group. Whereas concomitant treatments resulted in a marked rise *(p* < *0.05)* in the level of SOD and CAT in addition to total GSH, reduced GSH and GSH/GSSG ratio levels (by 23.10%, 30.94%, 14.22%, 44.905% and 180.2%, respectively) as linked to the rotenone-treated group.

There was no statistical difference between healthy rats and CBE-treated rats in GSSG concentration. Conversely, a statistical increment *(p* < *0.05)* in the GSSG concentration was observed in rotenone-treated rats (by 138.79%) relative to the control healthy group. Moreover, the combined treatment caused a significant drop in GSSG concentration (by 47.96%) compared to the rotenone group (Fig. 2J).

### Effect of *Chasteberry* extract (CBE) against rotenone-induced sperm count, motility, and morphologic changes in rats’ testes

**Fig. 3:**
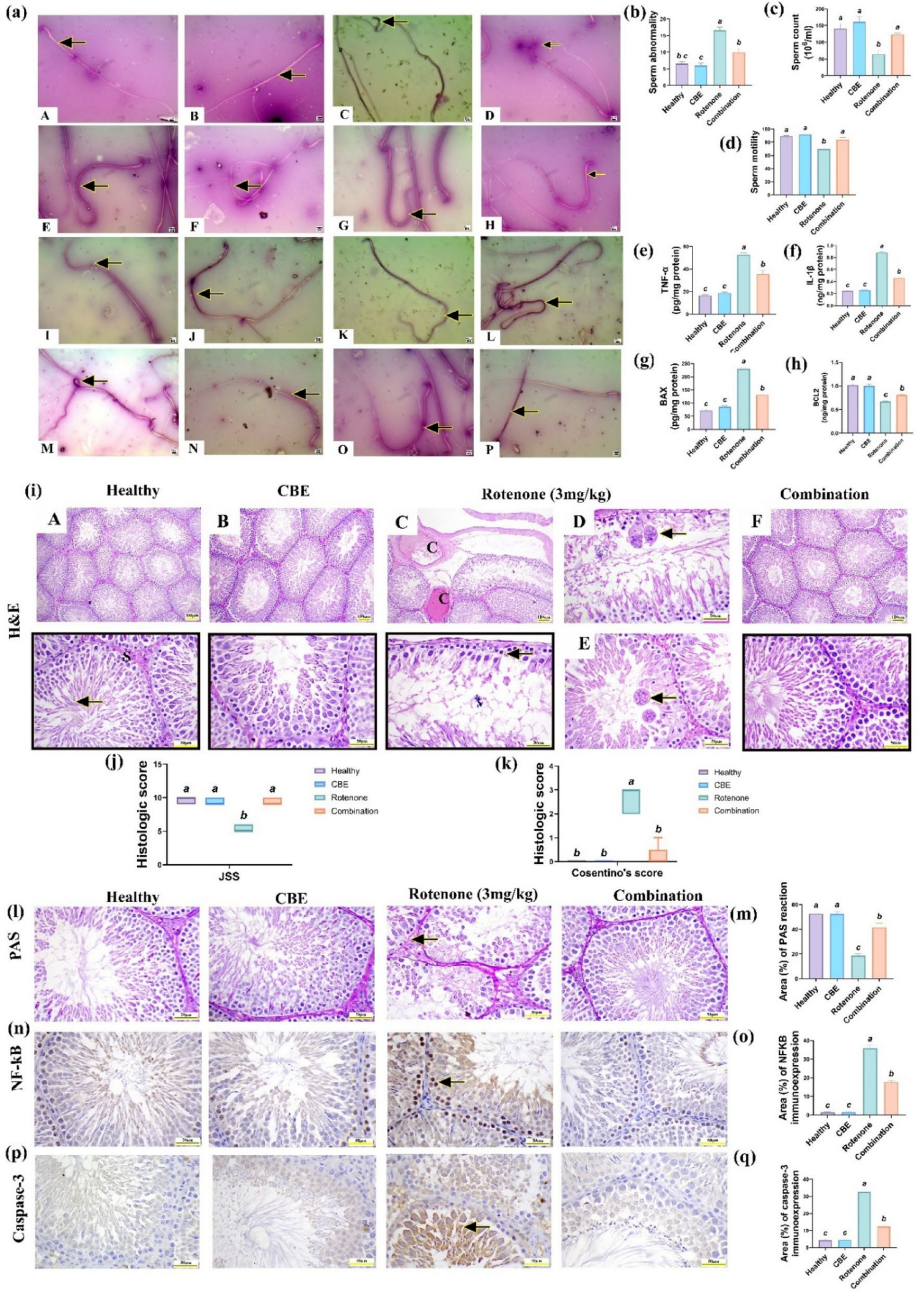
Effect of *Chasteberry* extract (CBE) against rotenone-induced sperm abnormalities, histopathologic changes, and apoptosis in rats’ testes. (**a**) Morphologic sperm abnormalities A&B: healthy and CBE treated groups, C–O: rotenone treated group, P: combination group showing normal sperm morphology. *Bar* = *20µm*. (**b**) Sperm abnormalities. (**c**) Sperm count (10^6^ × 10). (**d**) Sperm motility. (**e**) Tumor necrosis factor alpha (TNF-α, pg/mg protein). (**f**) Interleukin-1beta (IL-1β, ng/mg protein). (**g**) Bcl-2 Associated X (BAX, pg/mg protein). (**h**) B-cell leukemia/lymphoma 2 (BCL2, ng/mg protein). (**i**) Hematoxylin and Eosin (H&E) stained sections, (**A**) Healthy group showing typical sized seminiferous tubule lined by normal spermatogonia and central spermatozoa (S), (**B**) CBE group, (**C**) Rotenone group showing congested vessels with destruction of seminiferous tubules that only lined by spermatogonial cells (arrow), (**D**) Rotenone group showing spermatocytes giant cells, (**E**) Rotenone group showing spermatid giant cells, (**F**) Combination group showing restoration of tubular histoarchitecture. *Bar (A–C & F)* = *100µm and D, F & insets* = *50µm*. (**j**) Johnson spermatogenic histologic score (JSS). (**k**) Cosentino’s score. (**l**) Periodic acid Schiff (PAS) stained sections from different groups (Arrow indicates faint PAS staining in seminiferous tubules). (**m**) Area (%) of PAS staining. (**n**) Nuclear factor kappa beta (NF-κB) immunostaining in different groups (arrow indicating positive brown immunoreactivity of NF-κB. (**o**) Area (%) of NF-κB immunoreactivity. (**p**) Caspase-3 immunoexpression in different experimental groups (Arrow indicating positive cytoplasmic immunoexpression in spermatocytes). (**q**) Area (%) of caspase-3 immunoreactivity. *Bar* = *50µm*. Data was analyzed using one-way ANOVA followed by Tukey’s post hoc test. Histologic score was analyzed using the Kruskal–Wallis test, followed by Dunn’s test. Bars bearing different letters are significantly different at *p* < *0.05*.

Figure 3b indicated that no significant changes were recorded in the percentage of total sperm abnormalities between the healthy and CBE-treated groups. However, there was a significant increase *(p* < *0.05)* in the percentage of total sperm abnormalities (142%) in the group treated with rotenone relative to the healthy group. Notably, the combination group significantly *(p* < *0.05)* reduced the percentage of abnormal sperm by 37.5% relative to the group treated with rotenone alone.

As illustrated in Fig. 3c, no significant changes were recorded in total sperm count between the healthy and CBE-treated groups. In contrast, there was a significant reduction *(p* < *0.05)* of approximately 54% in total sperm count in the rotenone-treated rats relative to the healthy rats. While, co-administration of CBE and rotenone resulted in a marked increase *(p* < *0.05)* of 92% in total sperm count compared to the rotenone treated group.

As shown in Fig. 3d, no statistical variations were recorded in sperm motility between the healthy and CBE-treated groups. Conversely, rotenone treatment led to a significant decline *(p* < *0.05)* of 22.47% in progressive sperm motility when measured against the healthy group. However, the combination treatment of CBEand rotenone significantly *(p* < *0.05)* improved progressive motility by 21.16% versus the rotenone-treated group.

### Effect of *Chasteberry* extract (CBE) against rotenone-induced testicular inflammation and apoptosis in rats’ testes

As shown in Fig. 3e, f, there was no important variation between the healthy group and the CBE-treated group in the level of testicular proinflammatory cytokines (TNFα and IL1-β). Conversely, rotenone-intoxicated rats exhibited a significant *(p* < *0.05)* increase in the levels of TNFα and IL1-β (by 218.18% and 259.35%, respectively) comparatively to healthy group. While, the mixed group exhibited a significant *(p* < *0.05)* decline of testicular TNFα and IL1-β levels (by 32.116% and 47.9%) in contrast to the rotenone-intoxicated group.

Concerning the apoptotic markers, no statistical differences were reported between the healthy group and the CBE-treated group in BAX and Bcl2. Conversely, there was a marked *(p* < *0.05)* upsurge in the level of proapoptotic BAX (by 215.45%) with a significant decline of antiapoptotic Bcl2 level (by 35.2%) in rotenone- treated group versus the healthy group. However, the combination group presented a significant *(p* < *0.05)* decline of BAX levels (by 42.8%) with a significant rise in the level of Bcl2 (by 21.257%) in contrast to the rotenone-intoxicated group (Fig. 3g, h).

### *Chasteberry* extract’s protective effects against rotenone-induced testicular damage in rats.

Microscopic inspection of rats’ testes in healthy and CBE-treated groups presented distinctive interstitial Leydig cells and normal-sized seminiferous tubules with various developmental stages of spermatozoa (Fig. 3iA, iB, respectively). In contrast, rotenone-treated rats showed vascular congestion with degenerated seminiferous tubules lined only by spermatogonia cells, forming multinucleated spermatocytes and spermatid giant cells (Fig. 3iC–E). Furthermore, testicular tissue of the combination group showed an improvement in testicular histoarchitecture with massive intraluminal spermatozoa (Fig. 3iF). Statistical analysis of Johnsen’s histologic grading showed that rotenone-treated animals had a considerable decline in Johnsen’s score relative to healthy and CBE treated groups. Furthermore, this score significantly upregulated in the testicles of the combination group equated to the rotenone-only group (Fig. 3j). Cosentino’s score exhibited a significant increase in degenerative changes of rotenone-only-treated animals compared to control and CBE groups. However, the combination group showed significant decline in Cosentino’s score relative to rotenone-treated animals (Fig. 3k).

Microscopic examination of PAS stained testicles from different experimental groups (Fig. 3l). PAS staining of rats’ testicles revealed strong positive PAS in the basement membrane of seminiferous tubules and acrosomal cap in control and CBE-treated groups. Conversely, rotenone-treated animals showed mild PAS in the seminiferous tubule basement membrane. The combination group showed moderate to strong PAS Within the basement membrane of the seminiferous tubules. Semi-quantification of PAS area% indicated a significant reduction in PAS staining in the rotenone-treated animals relative to control and CBE-treated groups. The combination group showed was significantly upregulated PAS area% compared to rotenone-treated animals (Fig. 3m).

### Effect of *Chasteberry* extract (CBE) against rotenone-induced immunohistochemical changes in rats’ testes

Figure 3n displayed NF-κB immunoreactivity in different experimental groups. NF-κB immunoreactivity in rats’ testicles revealed negative nuclear immunoreactivity in healthy and CBE-treated groups. Conversely, rotenone-treated rats showed an intense nuclear immunoreactivity compared to healthy rats. The combination group showed a mild to negative NF-κB immunoreactivity. Semi-quantification of NF-κB immunoreactivity area% revealed a significant increase in the rotenone-treated group relative to healthy and CBE-treated groups. While the combination group showed a significant decline in NF-κB immunoreactivity area% compared to rotenone-treated animals (Fig. 3o).

Figure 3p displayed caspase-3 immunoreactivity in different experimental groups. Caspase-3 immunoreactivity in rats’ testicles revealed negative to mild immunoreactivity in control and CBE-treated groups. Conversely, rotenone-treated animals showed an intense cytoplasmic immunoreactivity, particularly in spermatocytes. The combination group showed a mild to negative caspase-3 immunoreactivity. Semi-quantification of caspase-3 immunoreactivity area% revealed a significant increase in the rotenone-treated animals relative to healthy and CBE-treated groups. The combination group showed a significant downregulation in the area% of caspase-3 immunoreactivity compared to rotenone-treated animals (Fig. 3q).

### Effect of *Chasteberry* extract (CBE) versus rotenone-induced oxidative damage and modification in mitochondrial dynamics in rats’ testes

**Fig. 4:**
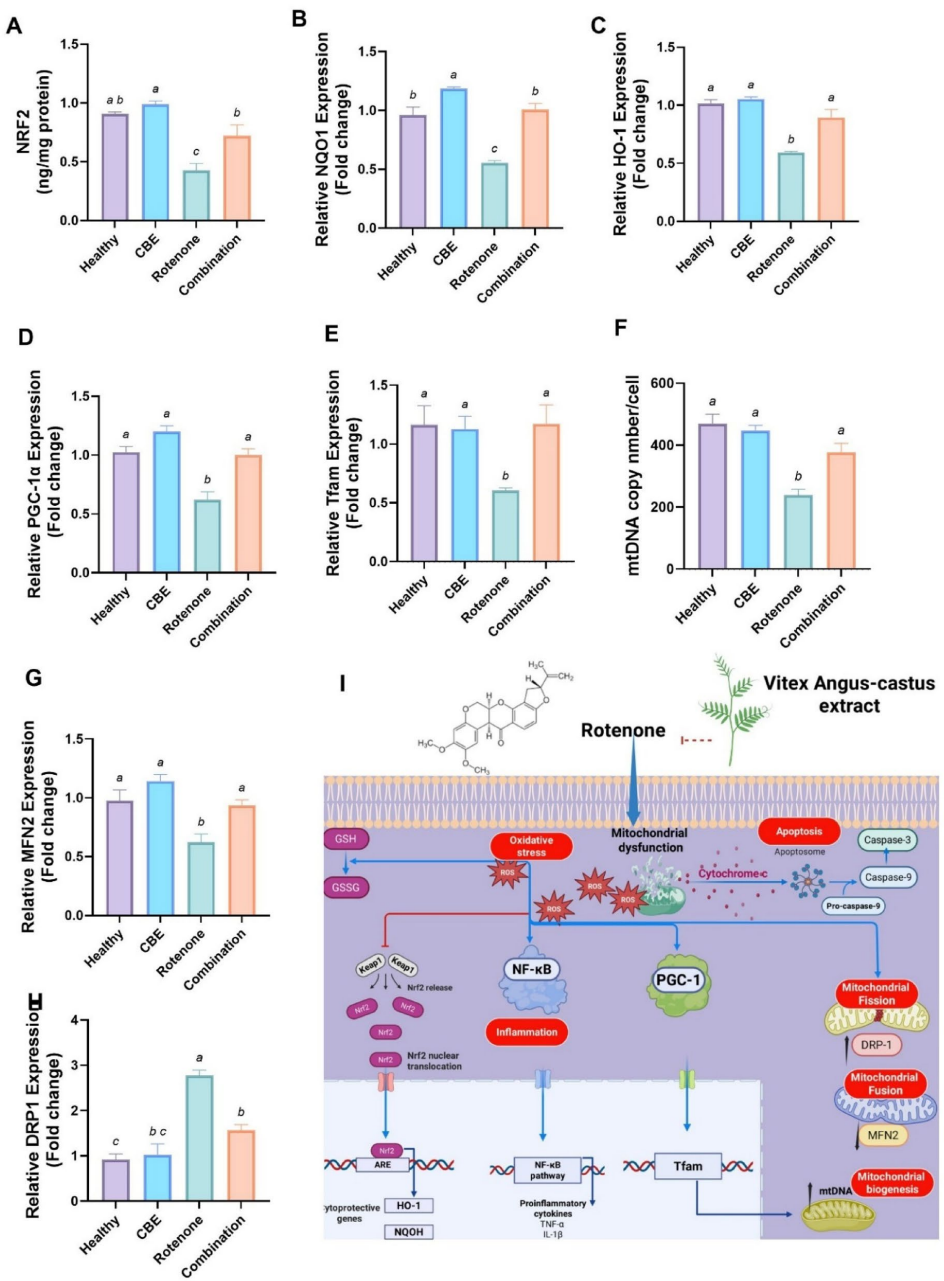
Effect of *Chasteberry* extract (CBE) against rotenone-induced oxidative stress and alterations in mitochondrial biogenesis and dynamics in rats’ testes. (**A**) Nuclear factor erythroid 2-related factor 2 (NRF2, ng/mg protein). (**B**) Relative gene expression of quinone oxidoreductase (NQO1). (**C**) Relative gene expression of heme oxygenase-1 (HO-1). (**D**) Relative gene expression of peroxisome Proliferator-Activated Receptor-γ Coactivator 1-α (PGC1α). (**E**) Relative mitochondrial transcription factor A (Tfam) gene expression. (**F**) mtDNA copy/cell. (**G**) Relative gene expression of mitofusin (MFN2). (**H**) Relative gene expression of Dynamin-related protein 1(DRP1). (**I**) Mechanistic action of CBE against rotenone-induced testicular toxicity. Data expressed as Mean ± SE (n = 5). Data was analyzed using one-way ANOVA followed by Tukey’s post hoc test. Bars bearing different letters are significantly different at *p* < *0.05*.

Regarding oxidative stress biomarkers, gene expression of NRF2, NQO1 and HO1 didn’t show significant changes *(p* > *0.05)* between the healthy rats and CBE-treated rats. In contrast, rotenone-intoxicated groups revealed an observable *(p* < *0.05)* downregulation in NRF2, NQO1 and HO1 mRNA transcripts by 52.86%, 42.29% and 41.42%, respectively if compared to the control group. Conversely, the combination group displayed a significant *(p* < *0.05)* increase in expression of NRF2, NQO1 and HO1 mRNA transcript by 68.22%, 81.95%, 50.48%, respectively when versus the rotenone group (Fig. 4A–C).

Regarding mitochondrial biogenesis biomarkers, gene expression of PGC1-α, Tfam and mtDNA copy number didn’t show significant changes *(p* > *0.05)* between the control rats and CBE-treated rats. In contrast, rotenone intoxicated groups revealed an observable *(p* < *0.05)* downregulation in expression of PGC1-α, Tfam and mtDNA copy number by 39.57%, 48.10% and 49.34%, respectively if compared to the healthy group. Conversely, the combination group showed a significant *(p* < *0.05)* increase in expression of PGC1-α, Tfam and mtDNA copy number transcript by 61.61%, 94.03%, 58.57%, respectively when versus the rotenone group (Fig. 4D–F).

Regarding mitochondrial dynamics, there is no observable difference between healthy rats and CBE-treated rats in both MNF2 and DRP1 gene expression. Conversely, rotenone administration exhibited a significant *(p* < *0.05)* downregulation in MNF2 gene expression (by 36.27%) and upregulation in DRP1 mRNA transcript (by 203.06%) in comparison with the healthy group. Furthermore, combined treatment displayed a significant *(p* < *0.05)* upregulation in MNF2 gene expression (by 50.48%) and downregulation in DRP1 mRNA transcript (by 43.44%) versus the rotenone-treated group (Fig. 4G, H).

## Discussion

Environmental contaminants, mainly pesticides, are recognized contributors to male infertility, primarily through their capacity to induce oxidative stress and disrupt hormonal balance^68^. Rotenone is a pesticide known for its potential toxicity, primarily because it strongly inhibits mitochondrial complex I. This interference disrupts the production of adenosine triphosphate, which is vital for cellular energy. More significantly, it leads to oxidative damage. These combined effects ultimately impair both the quality and quantity of sperm^69,70^. Additionally, rotenone-induced brain toxicity has been extensively studied, but its toxicity to the testes has received less attention. Also, there are little available studies on the appropriate medicine to counteract or lessen its adverse effects on the testes^71^. Thus, this study is designed to investigate whether CBE has a protective effect against the damage caused by rotenone in the testicles of male rats.

Exposure to pesticides can result in male sexual dysfunction and hormonal imbalances, including low testosterone levels^72^. Our data revealed a notable decrease in the blood levels of sex hormones (FSH, LH, and testosterone) in rotenone- treated rats. These hormonal changes suggest that rotenone-induced oxidative stress may disrupts the hypothalamic-pituitary–gonadal (HPG) axis, which is critical for normal spermatogenesis and reproductive function^27,73^. Also, this might be attributed to rotenone metabolites-induced disruption of the testicular gonadal-axis (male Leydig and Sertoli cells), which regulates the secretion of hormones^74^. Moreover, it might be ascribed to the degeneration of Leydig cells, that responsible for the production of testosterone^71^. Conversely, CBE administration effectively reversed rotenone-induced sex hormonal disturbances, suggesting a protective effect on both the testicular environment and the endocrine system. Results showed a noteworthy rise in levels of FSH, LH and testosterone in the mixed group treated with CBE + rotenone. Our findings are consistent with the results of Olaolu, Ajibola^44^. This effect is primarily attributed to the antioxidant properties of CBE, preventing rotenone-induced oxidative stress and neuroendocrine imbalance. Consequently, antioxidant supplementation can activate the hypothalamic pituitary axis (HPG) axis^75^. These results suggest that CBE can counteract rotenone-induced endocrine disruption and protect reproductive hormone homeostasis.

Oxidative stress is a major factor contributing to male infertility^76^. Our study showed that rotenone administration to male rats significantly amplified testicular oxidative stress. Results showed a significant increase in the testicular MDAand GSSG concentration along with a significant decline in SOD, catalase, total GSHand reduced GSHlevels. These changes indicate elevated oxidative stress and impaired antioxidant defense in the testes. Similar results were observed by Jain, Hasan^71^, Subramaniam, Vergnes^77^, Also results obtained by Umer, Sharif^48^ confirmed that rotenone administration to mice increased oxidative stress (MDA) with decreased antioxidant capacity (GSH, catalase and SOD) in brain tissues. As reported by Akintunde, Farai^27^, rotenone is broken down by a hydroxylation process that largely takes place in the liver and produces a lot of water-soluble metabolites. This makes it possible to pass easily through the testicular biological membranes barriers. Rotenone undergoes metabolic conversion by the microsomal CYP-450 enzyme system, yielding several highly reactive intermediates like rotenolone I, rotenolone II, 8′-hydroxyrotenone, and 6′,7′-dihydro-6′,7′-dihydroxyrotenone. These detrimental metabolites then bind with intracellular proteins, triggering lipid peroxidation and inducing oxidative stress^78^. Rotenone disrupts the electron transport chain within the mitochondria of testicular cells. By inhibiting complex I of this respiratory chain, it impairs oxidative phosphorylation, resulting in a reduced production of adenosine triphosphate (energy) in these tissues^77^. Conversely, co-administration of CBE extract ameliorated rotenone-induced oxidative stress in rat testes. Our data showed a significant decline in oxidative biomarker levels with a noteworthy upsurge in the levels of antioxidant biomarkers in the combined group. These results come in line with Ibrahim, El-Newary^46^. The antioxidant effect of CBE might be due to its phenolic acids and their extracts as flavonoids, tannins, and iridoids, each having its own antioxidant effect^79^. Thus, flavonoids and tannins may be the chemicals that give CBE its antioxidant properties^80^. Moreover, the total ability of CBE to fight oxidative stress and protect cells from harm may be improved by the cumulative effect of this variety of derivatives^81^.

Our findings demonstrated that rotenone administration significantly impaired several key sperm parameters, including total sperm count, motility, and abnormality. These changes might have occurred due to rotenone-induced oxidative stress by an upsurge in the production of hydrogen peroxide^82^. Rotenone also damages mitochondria, leading to cell death through apoptosis. This occurs because rotenone disrupts the cell’s energy production and increases the generation of ROS^83^. Then, ROS attack the sperm’s plasma membrane, impairing its fluidity. This, in turn, causes the sperm to lose its ability to move and damages the DNA within its mitochondria^84^. These results are consistent with Davila’s observations, who showed that exposure to rotenone led to sperm death and negatively affected both their ability to move (motility) and the structural soundness of their membranes (membrane integrity)^82^. Conversely, the combined treatment of CBE extract with rotenone pointedly ameliorated the harmful effects of rotenone on semen parameters. Rats receiving CBE in conjunction with rotenone exhibited marked improvements in sperm quantity and motility, as well as a reduction in the percentage of sperm abnormality. These beneficial effects may be attributed to the powerful antioxidant effect of CBE, which includes free radical scavenging and protection against lipid peroxidation and mitochondrial dysfunction. Bioactive compounds present in CBE, such as flavonoids and iridoid glycosides, are known to exert antioxidant and endocrine-modulating effects^80^.

Concerning the pro-inflammatory cytokines, our data showed a remarkable increase in the level of testicular TNF-α and IL-1β protein levels, besides an intense NF-кB immunoreactivity in the testicular tissue of the rotenone-treated group. This might be due to rotenone-induced oxidative stress by increased production of ROS, which triggers an inflammatory reaction^46^. Besides, numerous studies have demonstrated that rats receiving rotenone therapy significantly increased NF-кB activation^85,86^. Then the activated NF-кB translocate to the nucleus, where it starts many proinflammatory factors, including IL-1β, and TNF-α^87^. Our findings come in line with those of Alzarea, Afzal^88^, Ishola, Awogbindin^89^, Bakhsh, Abu-Baih^90^. Moreover, Morales-Del-Rio, Gutiérrez-Lomelí^91^ stated that rotenone administration in mice induced inflammation and increased concentrations of proinflammatory cytokines (IL-1β and TNF-α) in brain tissue. On the contrary, the current data showed that the mixed treatment of rotenone-intoxicated rats with CBE extract significantly reduced the testicular TNF-α and IL-1β levels. This could be illuminated by the prominent anti-inflammatory effect of CBE. A number of secondary metabolites that were separated from CBE, including p-hydroxybenzoic acid, methyl 3,4-dihydroxybenzoate, and 3,4-dihydroxybenzoic acid, demonstrated notable anti-inflammatory efficacy^92^. Also, the antioxidant capacity of CBE extracts is partly due to the presence of vitexin^91^. Additionally, Borghi, Carvalho^93^ noted that vitexin is a flavonoid that prevents the generation of pro-inflammatory cytokines. Our data are consistent with those of Ibrahim, El-Newary^46^, Ulusoy, İnal^94^.

Our results confirmed that rotenone treatment induced apoptosis in the testicular tissues. Results showed a significant rise in the level of BAX and caspase-3 with a significant decline in the Bcl2 level in the testicular homogenate of rats only treated with rotenone. It has been noted that increased BAX expression activates the intrinsic apoptotic pathway^95^. Our results come in line with those of Bakhsh, Abu-Baih^90^, Khosravi, Hojati^96^. Another study by Tian, Cao^13^ showed that in mouse testes and TM3 cells, rotenone caused apoptosis via endoplasmic stress and induced activation of the PERK-EIF2α-CHOP pathway. Moreover, our data showed that mixed treatment of rotenone with CBE ameliorated rotenone-induced apoptosis by decreasing the level of BAX with increasing the Bcl-2 level in testicular homogenate. CBE’s ability to prevent apoptosis may be attributed to its potent antioxidant and anti-inflammatory properties, as has been previously demonstrated^94^.

The gene NFE2L2 encodes for the transcriptional factor nuclear factor (erythroid-derived 2)-like 2 (Nrf2), which belongs to the Cap‘n’collar family of transcription factors. Nrf2 is responsible for regulating the baseline and stress-induced expression of more than 250 genes that contain the antioxidant response element (ARE) sequence. ARE is a specific DNA sequence located in the promoter region of numerous genes involved in cellular protection, particularly those coding for antioxidant and phase II detoxification enzymes^97^. These genes form a protective response against oxidative agents, including those that encode for HO-1 and NQO1. Nrf2 activation, which may be regulated via PGC-1*α,* increases the expression of antioxidant enzymes such as NQO1, HO-1, and others. Therefore, mitochondrial function and ATP synthesis are improved, and the oxidative damage can be prevented^97–99^. Darawsha, Trachtenberg^100^ observed that human dermal fibroblast exposure to rotenone resulted in mitochondrial dysfunction, subsequent ATP levels and respiration reduction, followed by increased production of mitochondrial and cytosolic ROS, triggering oxidative stress. Additionally, rotenone inhibits the electron transport complex in mitochondria, causing oxidative stress^101,102^. Flavonoid constituent of CBE has proven to have a strong exerting vital role in activating the Nrf2 signaling pathway. Subsequently, Nrf2 translocate into the nucleus, activating the previously mentioned ARE-related genes^103^. Thus, the protective effect of the combination of CBE and rotenone can be attributed to subsequent antioxidant response element (ARE/Nrf2) activation, which is observed in upregulation of the NQO1 and HO-1 proteins.

Our results demonstrated that PGC-1*α* and Tfam were highly expressed in combination groups than the group that received rotenone only. PGC-1*α* is considered the primary controller of mitochondrial biogenesis. Recently, PGC-1*α* has been linked to numerous inflammatory and metabolic diseases^104^. Nevertheless, it plays an integral role in regulating mitochondrial ROS production and detoxification, oxidative stress, and metabolic pathways in various tissues^105^. In the same context, the activation of PGC-1*α* controls both mitochondrial and nuclear genes that are crucial for mitochondrial biogenesis. It achieves this by activating various transcription factors, such as nuclear respiratory factors (NRF-1 and NRF-2). These, in turn, boost the TFAM, which then stimulates the transcription and replication of mtDNA. Moreover, the interaction between PGC-1*α* and PPAR stimulates OXPHOS gene expression in the nucleus and mitochondria, promoting mtDNA replication^104^. Noteworthy, PGC-1*α* and the stress response factor NF-*κ*B, a stress response factor that governs the immune response, have a mutually influential relationship. During periods of inflammation, NF-*κ*B diminishes both the expression and activity of PGC-1*α.* This reduction in PGC-1*α* activity subsequently leads to decreased expression of antioxidant genes, resulting in oxidative stress. In the same line, Peng, Tao^106^ reported that rotenone-induced neurotoxicity is associated with a reduction in the expression of PGC-1*α* in rats, which altered the mitochondrial biogenesis process and resulted in mitochondrial mass decrease. Similarly, Xiao, Dong^107^ noted that PGC-1*α* can be involved in repairing the damage induced by rotenone in murine PC12 cells by interacting with repair enzyme like EXOG*.* Moreover, Heo, Sun^20^ suggested that mitochondrial dysfunction triggered by rotenone prevented the maturation of porcine oocytes, which can be attributed to the downregulation of PGC-1*α.*

According to our data, rotenone injection resulted in alterations in the mRNA transcript of mitochondrial dynamics genes, as it increased mitochondrial fission (DRP1) and decreased mitochondrial fusion (MFN2). Aldridge, Benson^108^ reported that a lack of sufficient MFN2 leads to fragmented mitochondria, a decrease in their membrane potential, and a weakened ability to perform oxidative phosphorylation. These issues, in turn, negatively impact cellular respiration and growth. In addition, meiotic arrest and male sterility in knockdown mice of the gene encoding the MFN2 were reported by Tilokani, Nagashima^109^, Watanabe, Chuma^110^, Huang, Gao^111^. Similarly, knockdown mice of MFN1 and/or MFN2 showed abnormal spermatogenesis and male infertility. Oxidative stress occurs when there’s an imbalance between the creation of ROS and the body’s antioxidant defenses. This imbalance promotes mitochondrial fission by encouraging the movement of DRP1^109,112^. Labrousse, Zappaterra^113^ stated that DRP1 overexpression resulted in mitochondrial fragmentation, while a mutation in the same gene can produce mitochondrial tubularization. A study by Li, Zimmerman^114^ showed that rotenone produced an increase in mitochondrial fragmentation, as shown by mitochondrial staining and morphological examination, and increased the protein expression of mitochondrial fission markers, DRP1 and FIS1, in murine neuronal HT22 cells. This came in accordance with 100, which observed that an increased level of DRP1 resulted in alteration of mitochondrial fission/fusion dynamics due to rotenone treatment.

Amongst the proteins essential for mtDNA existence, one key protein, Tfam, which exerts an essential role in both packaging and transcribing of mtDNA^115^. Tfam coats mtDNA completely, resulting in bends at specific locations, enhancing the regulation of mitochondrial transcription and replication^116^. In the same line, Ylikallio, Tyynismaa^115^ reported that overexpression of Tfam increased mtDNA abundance in skeletal muscle tissue of mice up to sixfold. Peng, Tao^106^ observed the decrease in mtDNA number due to treatment with rotenone, which can be explained by the alteration in the level of expression of PGC-1*α* as a regulator of mitochondrial biogenesis and the level of DRP1 and MFN2. Several lines of evidence indicate that the transcriptional coactivator PGC-1α serves as a key regulator of mitochondrial biogenesis in mammals. The transcriptional coactivator γ coactivator 1α (PGC-1α regulates genes that are associated with energy metabolism. PGC-1α interacts with the nuclear peroxisome proliferator-activated receptor (PPAR), which permits the interaction of PGC-1α with a number of transcription factors^67^. PGC-1α is not a traditional genomic reference for mtDNA copy number, which may be considered as a potential limitation; however, with our methodological and normalizing approaches, it has been applied in quantifying mtDNA copy number in our study. Similarly, it has been successfully applied and normalized as mtDNA reference gene in rat soleus and cardiac muscles^65^. In addition, specific primers were designed to ensure specific amplification of the target gene. Then, PCR conditions were optimized to ensure efficient PCR amplification and efficiencies between mtDNA and PGC-1α. We suggest future study including additional conventional reference genes for cross-validation.

Also, a decrease in mtDNA was observed in porcine oocytes treated with rotenone, which was a result of oxidative stress, disruption of the mitochondrial biogenesis mechanism, and decreased SIRT1, which lowered PGC-1*α* activity^20^*.* Our results suggested that increased mtDNA copy number due to administration of CBE either alone or in combination with rotenone can be attributed to the improvement of mitochondrial biogenesis, enhancement of mitochondrial fusion, and the role of the described genes in promoting mitochondrial replication. Although CBE showed protective effects against rotenone-induced testicular damage, the efficacy may be influenced by factors such as the administered dose, treatment duration, and the bioavailability of active constituents, which should be considered when interpreting these results and planning future studies.

This research offers new insights into how CBE protects male rats from rotenone-induced testicular damage by examining its impact on hormonal balance, oxidative stress, inflammatory responses, and sperm quality. Furthermore, the study elucidates a unique mechanism involving the regulation of mitochondrial dynamics (DRP1, MFN2) and biogenesis (PGC-1α, Tfam), alongside the activation of the Nrf2/ARE pathway, positioning CBE as a potential natural intervention for rotenone-related reproductive disorders.

## Conclusion

According to our research, rotenone injection induced testicular damage, as evidenced by a decline in sex hormones (FSH, LH, testosterone) and antioxidant indicators (SOD, catalase, total GSH, reduced GSH) with an increase in oxidative stress biomarkers (MDA, GSSG), proinflammatory cytokines (TNFα and IL-1β) and apoptotic biomarkers, as it increased the pro-apoptotic BAX and caspase-3 expression besides decline in the anti-apoptotic BCL2 protein level. Additionally, rotenone injection resulted in alterations in sperm count, motility and count besides upregulation in sperm abnormalities. Molecularly, rotenone decreased the antioxidant mRNA transcript of antioxidant genes (NRF2, NQO1, HO-1), mitochondrial biogenesis genes (PGC1α), Tfam, mitochondrial copy number), as well as induced alterations in mitochondrial dynamics, as increased mitochondrial fission (DRP1) and decreased mitochondrial fusion (MFN2). Conversely, the CBE-supplemented rotenone group lessened these negative effects and restored sperm morphology and count. Histopathological analysis further supported these findings, showing that the combination group’s testicular histoarchitecture had significantly improved while nuclear factor kappa B and caspase 3 immunoreactivity had decreased. Besides, it increased the PAS reaction in the seminiferous tubular membrane. Further studies are needed to explore the underlying mechanistic action behind CBE’s therapeutic effects in male infertility.

## Data Availability

All data generated or analyzed during this study are included in this published article.

## Abbreviations

ROS: Reactive oxygen species
FSH: Follicle-stimulating hormone
LH: Luteinizing hormone
BAX: BCL2 associated X, apoptosis regulator
BCL2: B-cell lymphoma 2
MFN2: Mitofusin-2
DRP1: Dynamin-related protein 1
SOD: Superoxide dismutase
CBE: *Chasteberry* extract
DMSO: Dimethyl sulfoxide
IACUC: Institutional Animal Care and Use Committee
PBS: Phosphate-buffered saline
TNF-α: Tumor necrosis factor-alpha
IL-1β: Interleukin-1β
H&E: Hematoxylin and Eosin
PAS: Periodic acid–Schiff
NF-κB: Nuclear factor kappa beta
PGC-1α: Peroxisome proliferator-activated receptor-gamma coactivator-1 Alpha
Tfam: Mitochondrial transcription factor A
DRP1: Dynamin-related protein 1
HO-1: Heme oxygenase 1
NQO1: NAD (P)H quinone dehydrogenase 1
HPG: Hypothalamic pituitary axis
ARE: Antioxidant response element
